# Clinical Features, Diagnosis, and Outcome of Encephalitis in French Guiana

**DOI:** 10.4269/ajtmh.18-0308

**Published:** 2018-12-17

**Authors:** Alexandre Roux, Stéphanie Houcke, Alice Sanna, Cyrille Mathien, Claire Mayence, Romain Gueneau, Geoffroy Liegeon, Gaelle Walter, Dabor Resiere, Narcisse Elenga, Géraldine Resin, Felix Djossou, Didier Hommel, Hatem Kallel

**Affiliations:** 1 Intensive Care Unit, Cayenne General Hospital, Cayenne, French Guiana;; 2 Regional Authority of Health, Cayenne, French Guiana;; 3 Infectious and Tropical Diseases Unit, Cayenne General Hospital, Cayenne, French Guiana;; 4 Intensive Care Unit, Fort de France University Hospital, Fort de France, Martinique;; 5 Pediatric Unit Cayenne General Hospital, Cayenne, French Guiana

## Abstract

The aim of our study was to describe the clinical features, the etiologies, and the factors associated with poor outcome of encephalitis in French Guiana. Our study was retrospective, including all cases of encephalitis hospitalized in the Cayenne General Hospital, from January 2007 to July 2017. Patients were included through the 2013 encephalitis consortium criteria and the outcome was evaluated using the Glasgow outcome scale at 3 months from the diagnosis of encephalitis. We included 108 patients, giving an approximate incidence rate of four cases/100,000 inhabitants/year. The origin of the encephalitis was diagnosed in 81 cases (75%), and 72 of them (66.7%) were from an infectious origin. The most common infectious causes were *Cryptococcus* sp. (18.5%) independently of the immune status, *Toxoplasma gondii* (13.9%), and *Streptococcus pneumoniae* (5.5%). In the follow-up, 48 patients (46.6%) had poor outcome. Independent risk factors associated with poor outcome at 3 months were “coming from inside area of the region” (*P* = 0.036, odds ratio [OR] = 4.19; CI 95% = 1.09–16.06), need for mechanical ventilation (*P* = 0.002, OR = 5.92; CI 95% = 1.95–17.95), and age ≥ 65 years (*P* = 0.049, OR = 3.99; CI 95% = 1.01–15.89). The most identified cause of encephalitis in French Guiana was *Cryptococcus*. The shape of the local epidemiology highlights the original infectious situation with some local specific pathogens.

## INTRODUCTION

Encephalitis is a life-threatening condition caused by an inflammation of the brain parenchyma, leading to potentially severe neurologic dysfunction.^1^ It is an important public health issue, with a worldwide incidence ranging from 1.5 to 7/100,000 inhabitants/year and a case fatality of 7%.^2^ Encephalitis is a serious condition which is at high risk of severe sequelae and social burden in the long-term outcome. Diagnosis is challenging, with heterogeneous clinical presentations and a large number of etiologies spanning from autoimmune conditions to infectious diseases. Bacterial and viral agents have mainly been identified as causative agents related to encephalitis. Occasionally, fungus can be at the origin of encephalitis, especially among immunocompromised populations.^3^

However, despite the recent advances in diagnosis tools,^4,5^ approximatively 50% of acute encephalitis remains of unidentified cause.^4,6–8^ Causative agents of encephalitis are subject to regional variability. Rapid identification of the cause is the key to introduce urgent appropriate therapeutics.^9^ Also, there is a need for constant revaluation of the epidemiology because of emerging causes and/or dissemination of new triggers.^2,10^

Although extensively studied worldwide, there are no published data on encephalitis in the Amazonian region. Indeed, Boucher et al.^2^ performed a literature search on Medline database and did not find any study from South America.

In this work, we aimed to describe the clinical features, the etiologies, and the factors associated with poor outcome of encephalitis in patients admitted to the Cayenne General Hospital in French Guiana.

## PATIENTS AND METHODS

Our study is retrospective including all patients with a diagnosis of encephalitis admitted to the Cayenne General Hospital from January 2007 to July 2017. Our hospital is a 510-bed general center that serves as a first-line medical center for an urban population of 150,000 inhabitants and as a referral center (with the only intensive care unit (ICU) in the region) for a larger population coming from all French Guiana.

Cayenne is the regional capital of French Guiana, which is located on the North Atlantic coast of South America. It has borders with Brazil and Suriname. Its area is 83,534 square kilometers, with an estimated population of 254,000 people in 2014. The land is unequally inhabited, with most of the population living on the coastline, when a minority lives in the inside and remote villages. French Guiana is home to many unique and important ecosystems. Equatorial rainforests cover 95% of the territory and expose to a wide range of various infectious diseases.

In our study, we have divided the territory of French Guiana into two areas. The urban area is called the “coastline” with a road access to Cayenne. The journey lasts less than 3 hours by the road, whereas the “inside” are remote areas with no road access to Cayenne. These areas are reachable only by the airs or by the rivers, with at least 2 days journey for some of them.

### Patients and data sources.

Medical charts from all patients hospitalized for encephalitis, encephalomyelitis, and/or meningoencephalitis during the study period were identified using the Cayenne General Hospital database with the International Classification of Diseases, 10th edition.

In our hospital, informatised medical files date from 2008. So, for the first year of the study (2007), data were collected from the medical files (and not informatised files).

### Data collection and definitions.

Epidemiological, clinical, therapeutic data, complementary examinations, and outcomes were collected by A. R. (emergency disease specialist) and were reviewed by two raters blinded to the outcome: H. K. (intensivist) and F. D. (infectious disease specialist) to assess the diagnosis according to the following definitions:

Encephalitis from infectious and noninfectious origin and of any age was defined according to the Consensus Statement of the International Encephalitis Consortium criteria.^1^ The diagnosis of infectious encephalitis was confirmed when the pathogen was found in the cerebral spinal fluid (CSF), probable if the pathogen was found in the serum or if there was a seroconversion or a polymerase chain reductase (PCR) detection in the CSF, possible if there was a seroconversion in the serum, and clinical if no microbiologic confirmation was found but a combination of epidemiologic and clinical features, imaging findings, and biochemical analysis results strongly evocative of a disease and a negative result on a poorly sensitive test.^6^

Noninfectious encephalitis, which presents like infectious encephalitis, is divided into three subgroups: 1) paraneoplasic associated to intracellular antigens; 2) autoimmune with auto antibodies to extracellular epitopes of ion channels, receptors, and other associated proteins, such as the N-methyl-D-aspartate receptors (NMDAr); and 3) other forms of autoimmune encephalitis with antigens less clearly established, such as lupus cerebritis or acute disseminated encephalomyelitis (ADEM).^11^

The diagnosis of human immunodeficiency virus (HIV)-related acute encephalitis was based on a clinical, biological, and radiological bundle of arguments, as described in the HIV-associated neurological disorders. In this condition, 1) CSF often shows lymphocytic pleiocytosis with a possible detection and quantification of the HIV and 2) neuroimaging usually shows cortical atrophy and spread abnormalities of the sus-tentorial white matter.^12^

Immunocompromised state was defined as patients living with HIV having CD4^+^ < 200/mm^3^, having active malignancy, or using chronic immunosuppressive treatment.

Altered consciousness at hospital admission was evaluated clinically and by a Glasgow Coma Scale less than 15.

Glasgow Outcome scale was used to evaluate the neurological status at 3 months from the diagnosis of encephalitis. In our analysis, poor outcome was defined as death, vegetative state, or severe disability.^9,13^

### Data analysis.

Data were analyzed by using the Excel and SPSS program version 20 (SPSS, Chicago, IL). Continuous variables are expressed as mean and standard deviation or median and interquartile range (IQR). Categorical variables are expressed as number and proportion.

Differences between groups were assessed using Student’s *t*-test for continuous variables and χ^2^ test for categorical variables. Variables yielding a *P* value < 0.1 were entered into a multiple logistic regression model for the measurement of odds ratios and 95% confidence intervals with the diagnosis of “poor outcome” as a primary outcome. Statistical significance was set at the 5% level.

## RESULTS

**Figure 1.**
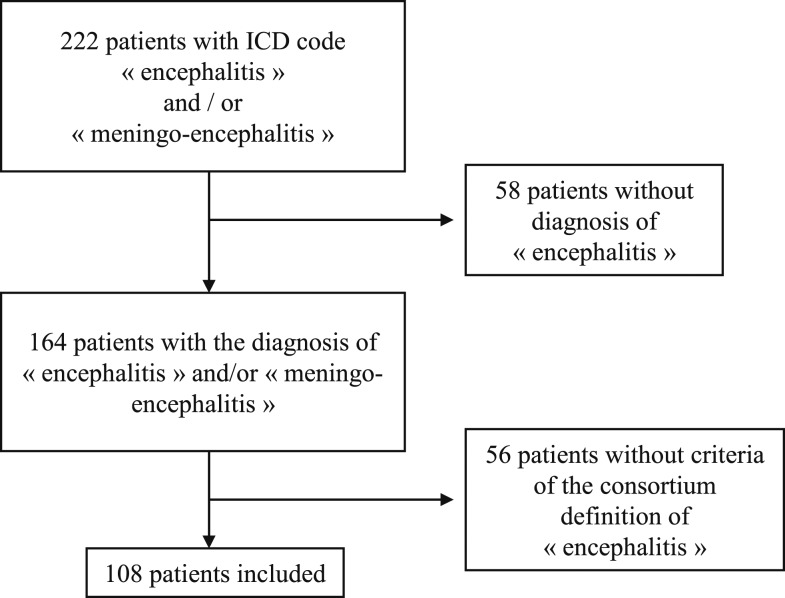
Study flowchart.

During the study period, 222 patients were likely to have encephalitis. Among them, 114 patients were excluded because they did not meet all the diagnosis criteria: 58 because of the lack of major criteria and 56 because they had less than two minor criteria. Overall, 108 patients were finally included in this study, giving an incidence rate of four cases/100,000 inhabitants/year. Figure 1 shows the study flowchart.

The mean age of our patients was 38.3 ± 20.5 years (extremes: min., 7 months and max., 80 years). Analysis did not show any seasonal variation. Fourteen patients (13%) were older than 65 years and 18 (16.7%) were pediatrics. Twenty-five patients (23.1%) came from the inside area. Sixty-eight (63%) patients had at least one chronic disease. The median time between the onset of symptoms and admission was 9 days (IQR = 2–14 days). Epidemiological data and the main symptoms at admission are reported in Table 1. The median length of hospital stay was 26 days (IQR = 11.7–53.5) and 45 (41.7%) patients were admitted in ICU.

**Table 1:** Epidemiological and initial clinical features of our population

Variable	Patients, *n* = 108
Age (years)	38.3 ± 20.5
Male/female ratio	2.3
Living in coastline area	83 (76.8)
Chronic diseases	
Hypertension	15 (13.9)
Diabetes	11 (10.2)
All HIV infection	45 (41.7)
Newly diagnosed HIV infection	11 (10.2)
Immunocompromised	49 (45.4)
Chronic intoxication*	12 (11.1)
Other medical history	22 (20.4)
Initial clinical symptoms	
Fever	77 (71.3)
Glasgow coma scale ≤ 8	30 (27.8)
Consciousness disorder	78 (72.2)
Behavioral disorder	63 (58.3)
Seizures	24 (22.2)
Headache	54 (50)
Meningeal signs	23 (21.3)
Focal signs	43 (39.3)
Intracranial hypertension	6 (5.6)

HIV = human immunodeficiency virus. Patient data are expressed as number (%) or mean ± standard deviation; Fever: T° > 38°C.

* Alcohol, tobacco.

Antibiotic treatment was used in 71 patients (65.7%), antiviral treatment was used in 43 patients (39.8%), and antifungals in 28 patients (25.9%). Corticosteroids and antiepileptic treatment were used in 24 patients (22.2%) each.

About complementary examinations, 92 (85.2%) patients had CSF analysis, 103 (95.4%) had a neuroimaging, and 31 (28.7%) had an electroencephalography (EEG). The results of those examinations are shown in Table 2.

**Table 2:** Complementary examinations

Variable	Nb*	Nb† (%) or mean (ext.)
CSF analysis	108	92 (85.2)
Time between admission and 1st CSF (days)	92	2.47 ± 14.5 (−82 to 93)
Lumbar puncture while hospitalization	108	80 (74.1)
Glucose level (mmol/L)	85	3 ± 1.9 (0–10.2)
Protein level (g/L)	86	2.1 ± 5 (1–37)
Lactate level (mmol/L)	43	4.3 ± 4.1 (2–24)
Lactate level ≥ 2.5 mmol/L	43	29 (67.4)
Gram stains	92	17 (18.4)
Culture	92	26 (28.3)
Neuroimaging	108	103 (95.4)
CT scan without IV contrast injection	103	55 (53.4)
CT scan with IV contrast injection	103	23 (22.3)
MRI	103	60 (58.3)
Abnormal neuroimaging	103	69 (67)
Abnormal MRI	60	43 (71.7)
EEG	108	31 (28.7)
Abnormal EEG	31	19 (61.3)
Diffuse slow activity on EEG	31	16 (51.6)

MRI = magnetic resonance imaging.

* Number of patients for whom the data were available.

† Number of patients for whom the data were positive.

The etiology of the encephalitis was identified in 81 cases (75%). It was of infectious origin in 72 cases (66.7%). The main causative agents were *Cryptococcus*, toxoplasmosis, and *Streptococcus pneumonia*. Among infectious causes, 38 (52.8%) were confirmed, 10 (13.9%) were probable, 13 (18.1%) were possible, and 11 (15.2%) were established on clinical arguments. The diagnostic probabilities according to the etiological origin among encephalitis of determinated causes are shown in Table 3. Etiologies and outcome in general population and in subgroups are shown in Table 4. We note that *Cryptococcus* is the most identified pathogen in all subgroups of immunocompetent, immunocompromised, and pediatric populations. *Toxoplasma gondii* was isolated only in immunocompromised adults.

**Table 3:** Diagnostic probability according to the etiological origin among encephalitis of determinated causes

	Confirmed	Probable	Possible	Clinical	Total
Virus	6 (35.3)	3 (17.6)	3 (17.6)	5 (29.4)	17 (100)
Varicella zoster virus	2 (50)	–	–	2 (50)	4 (100)
Human immunodeficiency virus	–	–	2 (50)	2 (50)	4 (100)
Herpes simplex virus	3 (100)	–	–	–	3 (100)
Progressive multifocal leukoencephalopathy	–	1 (50)	–	1 (50)	2 (100)
Dengue	–	–	1 (100)	–	1 (100)
Rabies	1 (100)	–	–	–	1 (100)
Tonate	–	1 (100)	–	–	1 (100)
Chikungunya	–	1 (100)	–	–	1 (100)
Bacterian	11 (68.7)	3 (18.7)	2 (12.5)	0	16 (100)
*Spneumonia pneumonia*	6 (100)	–	–	–	6 (100)
*Mycobacterium tuberculosis*	1 (33.3)	1 (33.3)	1 (33.3)	–	3 (100)
*Coxiella burnetii*	–	1 (50)	1 (50)		2 (100)
*Klebsielle pneumoniae*	2 (100)	–	–	–	2 (100)
*Listeria monocytogens*	1 (50)	1 (50)	–	–	2 (100)
*Haemophilus influenzae*	1 (100)	–	–	–	1 (100)
Parasitic	3 (17.6)	1 (5.9)	6 (35.3)	7 (41.2)	17 (100)
*Toxoplasma gondii*	2 (13.3)	–	6 (40)	7 (46.7)	15 (100)
*Trypanosoma cruzi*	–	1 (100)	–	–	1 (100)
*Isospora hominis*	1 (100)	–	–	–	1 (100)
Fungic	17 (77.3)	3 (13.6)	2 (9.1)	0	22 (100)
*Cryptococcus*	15 (75)	3 (15)	2 (10)	–	20 (100)
*Aspergillus*	1 (100)	–	–	–	1 (100)
*Histoplasma capsulatum*	1 (100)	–	–	–	1 (100)
Autoimmune	0	1 (14.3)	0	6 (85.7)	7 (100)
Acute disseminated encephaloMyelitis	–	–	–	6 (100)	6 (100)
Rasmussen	–	1 (100)	–	–	1 (100)
Other	1 (50)	0	0	1 (50)	2 (100)
Neurosarcoid	–	–	–	1 (100)	1 (100)
Adult T-cell leukemia/lymphoma	1 (100)	–	–	–	1 (100)
Total	38 (46.9)	11 (13.6)	13 (16)	19 (23.5)	81 (100)

**Table 4:** Causes of encephalitis in general population; pediatric, adult, immunocompetent, and immunocompromised population; area of living; and outcomes depending on the etiologies

	Adult No (%)	Pediatric No (%)	Immunocompetent No (%)	Immunocompromised No (%)	Coastline No (%)	Inside No (%)	Good outcome No (%)	Poor outcome No (%)	Total No (%)
Virus	15 (16.9)	2 (11)	6 (10.2)	11 (22.4)	13 (15.7)	4 (16)	7 (12.7)	9 (18.7)	17 (15.7)
Varicella zoster virus	2 (2.2)	2 (11)	2 (3.4)	2 (4.1)	4 (4.8)	–	3 (5.4)	1 (2.1)	4 (3.7)
Human immunodeficiency virus	4 (4.5)	–	–	4 (8.2)	4 (4.8)	–	3 (5.4)	1 (2.1)	4 (3.7)
Herpes simplex virus	3 (3.4)	–	1 (1.7)	2 (4.1)	1 (1.2)	2 (8)	1 (1.8)	1 (2.1)	3 (2.8)
Progressive multifocal leukoencephalopathy	2 (2.2)	–	–	2 (4.1)	2 (2.4)	–	–	2 (4.2)	2 (1.9)
Dengue	1 (1.1)	–	1 (1.7)	–	–	1 (4)	–	1 (2.1)	1 (0.9)
Rabies	1 (1.1)	–	1 (1.7)	–	1 (1.2)	–	–	1 (2.1)	1 (0.9)
Tonate	1 (1.1)	–	1 (1.7)	–	–	1 (4)	–	1 (2.1)	1 (0.9)
Chikungunya	1 (1.1)	–	–	1 (2)	1 (1.2)	–	–	1 (2.1)	1 (0.9)
Bacterial	14 (15.6)	2 (11)	12 (20.3)	4 (8.2)	14 (16.9)	2 (8)	7 (12.7)	8 (16.7)	16 (14.8)
*Spneumonia pneumonia*	5 (5.6)	1 (5.5)	5 (8.5)	1 (2)	5 (6)	1 (4)	2 (3.6)	4 (8.3)	6 (5.5)
*Mycobacterium tuberculosis*	3 (3.4)	–	2 (3.4)	1 (2)	2 (2.4)	1 (4)	1 (1.8)	1 (2.1)	3 (2.8)
*Coxiella burnetii*	2 (2.2)	–	2 (3.4)	–	2 (2.4)	–	2 (3.6)	–	2 (1.9)
*Klebsielle pneumoniae*	2 (2.2)	–	1 (1.7)	1 (2)	2 (2.4)	–	–	2 (4.2)	2 (1.9)
*Listeria monocytogens*	2 (2.2)	–	1 (1.7)	1 (2)	2 (2.4)	–	2 (3.6)	–	2 (1.9)
*Haemophilus influenzae*	–	1 (5.5)	1 (1.7)	–	1 (1.2)	–	–	1 (2.1)	1 (0.9)
Parasitic	17 (19.1)	0	2 (3.4)	15 (30.6)	17 (20.5)	-	12 (21.8)	5 (10.4)	17 (15.7)
*Toxoplasma gondii*	15 (16.8)	–	–	15 (30.6)	15 (18.1)	–	11 (20)	4 (8.3)	15 (13.9)
*Trypanosoma cruzi*	1 (1.1)	–	1 (1.7)	–	1 (1.2)	–	1 (1.8)	–	1 (0.9)
*Isospora hominis*	1 (1.1)	–	1 (1.7)	–	1 (1.2)	–	–	1 (2.1)	1 (0.9)
Fungic	20 (22.5)	2 (11)	8 (13.6)	14 (28.6)	13 (15.7)	9 (36)	9 (16.4)	12 (25)	22 (20.4)
*Cryptococcus*	18 (20)	2 (11)	8 (13.6)	12 (24.5)	11 (13.3)	9 (36)	7 (12.7)	11 (22.9)	20 (18.5)
*Aspergillus*	1 (1.1)	–	–	1 (2)	1 (1.2)	–	–	1 (2.1)	1 (0.9)
*Histoplasma capsulatum*	1 (1.1)	–	–	1 (2)	1 (1.2)	–	1 (1.8)	–	1 (0.9)
Autoimmune	2	5 (27.8)	7 (11.9)	0	7 (8.4)	-	5 (9.1)	1 (2.1)	7 (6.5)
Acute disseminated encephalomyelitis	1 (1.1)	5 (27.8)	6 (10.2)	–	6 (7.2)	–	4 (7.3)	1 (2.1)	6 (5.5)
Rasmussen	1 (1.1)	–	1 (1.7)	–	1 (1.2)	–	1 (1.8)	–	1 (0.9)
Other	2	0	1 (1.7)	1 (2)	2 (2.4)	-	1 (1.8)	1 (2.1)	2 (1.9)
Neurosarcoid	1 (1.1)	–	1 (1.7)	–	1 (1.2)	–	1 (1.8)	–	1 (0.9)
Adult T-cell leukemia/lymphoma	1 (1.1)	–	–	1 (2)	1 (1.2)	–	–	1 (2.1)	1 (0.9)
Unknown causes	20 (22.5)	7 (38.8)	23 (39)	4 (8.2)	17 (20.5)	10 (40)	15 (27.3)	12 (25)	27 (25)
Total	90	18	59	49	83	25	55	48	108 (100)

Overall, the case fatality rate was of 28.7%, including 29 patients (26.9%) who died during the hospital stay and two who died 3 months after the discharge. Ten patients (9.3%) were still hospitalized at three months and 64 patients (59.3%) left the hospital with a mean length of stay at 29 ± 20 days and were followed at the outpatient visit. Five patients were lost to follow-up. Poor outcome was observed in 48 patients (46.6%).

**Figure 2.**
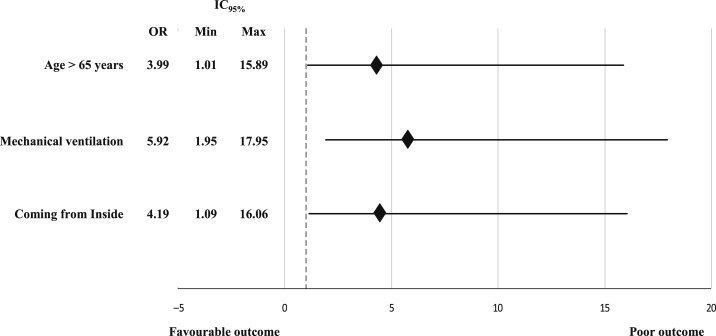
Forest plot showing the independent predictive factors of poor outcome in patients with encephalitis.

**Figure 3.**
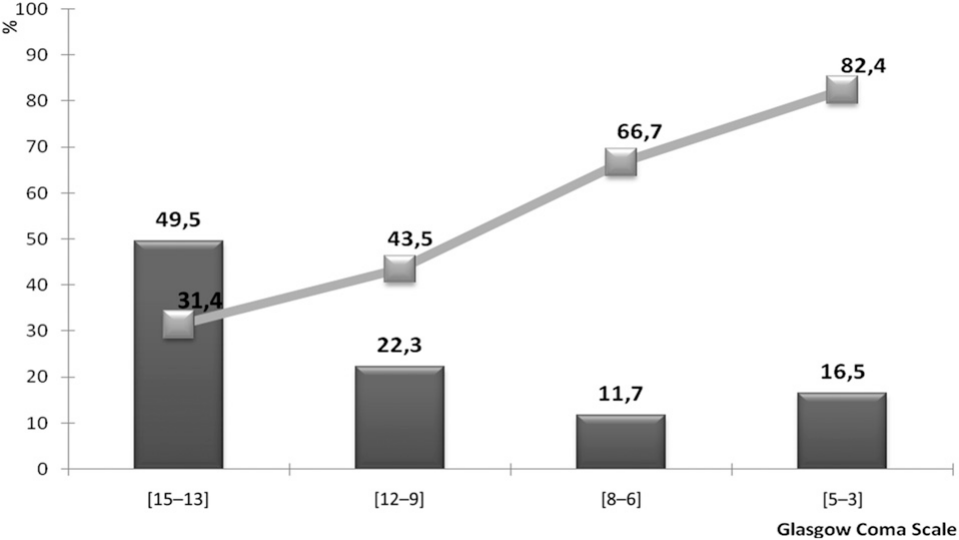
Glasgow coma scale at admission related to the poor outcome. Bars indicate the frequency of patients and the line indicates the frequency of poor outcome.

**Figure 4.**
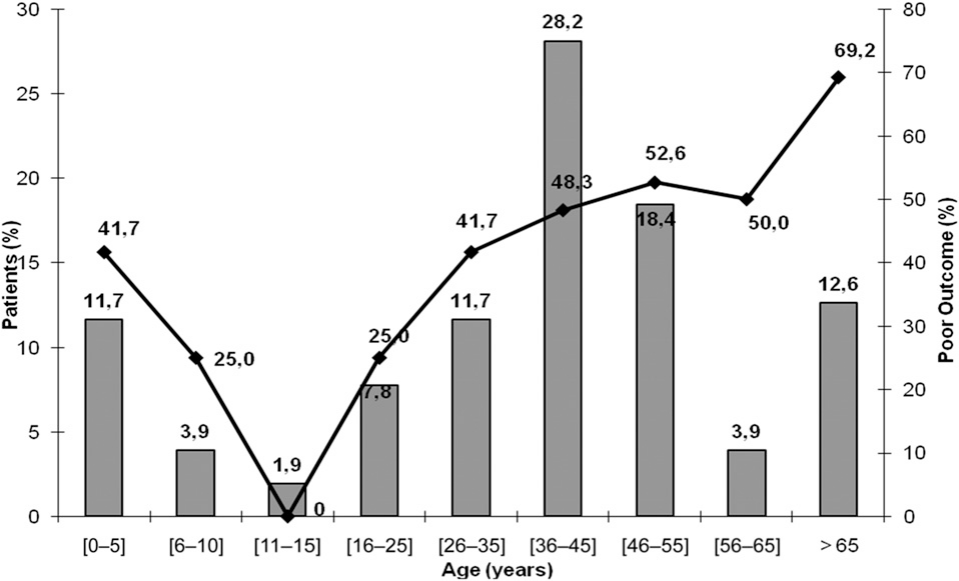
Poor outcome according to age in our patients. Bars indicate the frequency of patients and the line indicates the frequency of poor outcome.

Factor linked to poor outcome are shown in Table 5. In multivariable logistic regression model, factors associated with poor outcome were as follows: age older than 65 years, need for mechanical ventilation, and coming from the inside area of the region (Figure 2). Figures 3 and 4 show the outcome of our patients according to the consciousness level and to age.

**Table 5:** Factors associated with poor outcome in multivariable logistic regression model (for variables with *P* < 0.1 in univariate analysis)

	Univariate analysis			Multivariate analysis		
Variable	*P*	OR	CI 95%	*P*	OR	CI 95%
Age > 65 years	0.057	3.2	0.92–11.3	0.049	3.99	1.0–15.9
Area of living—from the inside	0.003	4.7	1.7–13.3	0.036	4.2	1.1–16.0
Hospitalisation in ICU	< 0.001	4.9	2.10–11.3	–	–	–
Mechanical ventilation	< 0.001	9.6	3.6–25.5	0.002	5.9	1.9–17.9
Consciousness disorder	0.025	2.9	1.1–7.3	–	–	–
Glasgow ≤ 8	< 0.001	5.8	2.2–15.4	–	–	–
Abnormal MRI	0.002	9.8	1.9–49.1	–	–	–
Diffuse slow activity on EEG	0.020	6.0	1.3–28.5	–	–	–
CSF lactacte > 2.5 mmol/L	0.004	9.3	1.7–49.7	–	–	–
Certain diagnosis of infection	< 0.001	5.6	2.2–13.8	–	–	–
PCR positive on CSF	0.008	3.7	1.4–20	–	–	–

OR= odds ratio. NB: all variables collected in our study were tested to outcome in univariate analysis, and the table shows only variables with *P* < 0.1.

## DISCUSSION

Our study shows that encephalitis is frequent in French Guiana and that the cause can be identified in up to 75% of cases. The most frequently identified infectious causes were cryptococcosis and toxoplasmosis. Poor outcome was observed in 46% of patients. The independent factors associated with poor outcome were “coming from inside area of the region,” need for mechanical ventilation, and age ≥ 65 years.

The mean age of our patients was 6 to 14 years. It was younger than what was observed in the United States and in mainland France.^6,7,14^ This result is explained by the French Guiana’s demography, among which 80.2% of the population is less than 44 years old, with a median of 28 years. Children represented 17% of our patients, similar to the France, U.S., and England studies (10–34%).^6,7,9^

Male–female ratio was 2.3, whereas it was 0.6–1.2^5^ in French and U.S. studies.^6,7^ This difference is neither not explained by the local demography nor by the high prevalence of HIV in our cohort. Indeed, sex ratio for HIV in French Guiana population is 1.^15^ However, it can be explained by the prevalence of fungal encephalitis in our cohort because *Cryptococcus* infects preferentially male.^16^

Notably, there is no significant seasonal change of encephalitis over years. We observed a higher incidence of encephalitis in 2009, 2010, and 2014. During 2009 and 2010, there were two dengue virus epidemics, and in 2014, there was a chikungunya epidemic.^17,18^ During the dengue epidemics of 2009 and 2010 there were only one case with encephalitis related to dengue virus, three ADEM, and seven cases of encephalitis with undetermined origin, among whom three had fatal issues. However, there was no spike of encephalitis in 2013, although there was also a dengue virus epidemic. So, there is no evidence of link between the occurrence of encephalitis and dengue epidemic. Nevertheless, in a meta-analysis about post-Dengue ADEM performed in Brazil, 6.8% of patients having dengue and neurological presentation developed ADEM.^19^ In the 2014 chikungunya epidemic, only one case of encephalitis caused by chikungunya was confirmed with a positive CSF PCR.

The mean hospital length of stay of our patients was twice more than that in others studies.^14^ This difference is explained by the lack of hospital structures and the absence of rehabilitation unit in French Guiana. Also, for a lot of our patients, social conditions of living do not permit to take them back home with medical assistance when needed.

Our patients had twice more comorbidities than the 30% observed by Mailles et al.^6^ because in our study, we included HIV patients (41.7% of comorbidities). In addition, among French regions, Guiana has one of the highest prevalence of diabetes, hypertension, obesity, and HIV.^15,20^ The HIV rate in our patients was substantially higher compared with the 6.3%, 7.7%, or 8.8% reported in other cohorts from France and the United States.^7,21,22^ The prevalence and the incidence of HIV recorded in French Guiana are 1.2–1.5% and 150–200 new cases/100,000 inhabitants/year.^15^

In our study, the cause of encephalitis was identified in 75% of cases. It was infectious in 66.6% of cases. This rate is higher than the one reported in the 2007 French prospective study on infectious encephalitis (52%).^6^ Worldwide, the causes of encephalitis are identified in 27.5–79% of cases.^2^

The first identified cause of encephalitis in our study was *Cryptococcus* sp., a rarely reported cause of encephalitis.^2,6,23,24^ Cryptococcosis is a common invasive fungal disease. It is responsible of one million infection cases and 650,000 deaths every year worldwide.^3^ In French Guiana, Debourgogne et al.^25^ conducted a retrospective study between 1998 and 2008, including 43 patients with cryptococcosis. In this study, 22 patients had neuro-meningeal expressions, and five of them were immunocompetent. In these five patients, the only pathogen found was *Cryptococcus gattii*. In our study, 20 cases were diagnosed with cryptococcosis encephalitis without identification of the subspecies. Over the 20 cases, eight did not have any comorbidity. This high prevalence of *Cryptococcus* can be explained by its easier identification in the CSF than other infectious pathogens, and it is probably related to the local ecology through the soil, trees, or to the local avifauna.^26^

The second cause of infectious encephalitis, in our study, was toxoplasmosis, which is exclusively isolated in patients living with HIV. This result is not surprising regarding the local prevalence of HIV infection in French Guiana and was commonly reported in studies including immunocompromised patients.^7,21^

The local shape of the epidemiology in our study highlights some tropical causes of acute infectious encephalitis such as *Coxiella burnetii* (Q fever), *Trypanosoma cruzi* (Chagas disease), Tonate virus, chikungunya, dengue virus, *Histoplasma capsulatum*, and rabies (RABV).

Q fever incidence in French Guiana is one of the highest in the world.^27^ The main risk factor is inhalation of aerosols of dusts. No link with classical sources of *C. burnetii* has been ever identified in French Guiana and a wild reservoir has been suspected.^27^ In our study, two cases of encephalitis were caused by *C. burnetii*. One of them was identified by PCR on the CSF and the other was diagnosed on positive blood serology.

Chagas disease (or American trypanosomiasis) is a widespread South American zoonosis transmitted not only by bloodsucking triatomine bugs (Hemiptera and Triatominae) but also by blood transfusion from infected donors and occasionally by transplacental route.^28^ The case of Chagas disease diagnosed in our cohort was confirmed by PCR on the CSF. Recently, a case of encephalitis was documented to Tonate virus (TONV), aIIIb subtype of the Venezuelan Equin Encephalitis complex.^29^ In our study, we emphasize also a case of fatal rabies which occurred in 2008. Several reservoirs of rabies virus are identified in Amazonian region but the major reservoir is vampire bats (*Desmondus rotundus*).^30^ Preventive vaccination against rabies is performed for at-risk population. It is worth to note that those last pathogens are not in the list of “main pathogens to consider depending on the context” for South America which appears in the International Encephalitis Consortium^1^ and in the literature research performed by Boucher et al.^2^

Autoimmune encephalitis is reported in 15–30% of cases with encephalitis in some studies.^8,10^ Despite having a similar clinical presentation to the infectious encephalitis, diagnosis of autoimmune encephalitis might be delayed because it depends on the time to antibody testing and to the response to immunotherapy.^31^ Early diagnosis of autoimmune encephalitis is essential. And, early introduction of steroids and/or immunosuppression in anti-NMDAr encephalitis is a predictor of good outcome.^32^

Over the past 10 years, we note an increasing detection of autoimmune encephalitis because of the improvement of diagnostic tools.^10^ In our study, we found seven cases (6.5%) of autoimmune encephalitis with six ADEM and one case of Rasmussen encephalitis. The diagnosis of Rasmussen encephalitis in our patient was confirmed after his transfer to a specialised center in Paris.

In the major English study,^8^ 23 cases of encephalitis were ADEM (11%), 80% of them were aged between 1 and 19 years, and 35% had serological evidence of recent infection.^8^ Indeed, pediatric population is predisposed to ADEM.^33^ In our study, we found six ADEM (5.5%). All of them were less than 16 years old. As for autoimmune encephalitis, in most patients, the diagnosis was confirmed after a transfer of the patients to a specialized center in mainland France. For all those reasons, we probably have an underestimation of the diagnosis of autoimmune encephalitis.

In our study, the inhospital mortality rate was 26.9%. This rate is higher than what is commonly observed.^6,8,21^ But, a higher rate of mortality of about 36% was observed in rural area of Central India.^34^ We think that our high rate of inhospital mortality can be explained by 1) the originality of the shape of our epidemiology, 2) the prevalence of immunocompromised status and comorbidities in our patients, 3) a socioeconomic condition of our population different from the one where major studies were lead, and 4) may be because of the delay between the onset of symptoms and hospital admission.

In our study, poor outcome was observed in 48 patients (46.6%). In previous studies, poor outcome was reported in 38% in immunocompetent population^13^ and in 56% in ICU population,^14^ and was associated to age ≥ 65 years and hospitalization in ICU.^9,14,35^ In our study, all these parameters were associated with poor outcome in the univariate analysis. Interestingly, we found a link between CSF lactate > 2.5 mmol/L and poor outcome in univariate analysis. Lactate level in CSF is known to make the difference between bacterial and aseptic meningitis^36^ or to assess the efficiency of the cure of bacterial meningitis.^37^ Furthermore, Mailles et al. reported a high diagnosis value of the lactate level in the CSF to assess bacterial origin of the encephalitis with specificity and a positive predictive value at 100% for a cutoff of 5.76 mmol/L.^6^ CSF lactate level reflects brain suffering which can be related to acute serious presentations such as seizure, brain hypoxia, and infection.^8^

In our patients, living in the remote areas of the territory was a significant independent factor linked to poor outcome assessed by multivariable analysis. Obviously, there is limited access to health care in some areas of the inside, and when needed, helicopter transportation is mobilized to transfer serious patients to our hospital. Even if those areas are inhabited by indigenous community from many ethnic groups, most of the patients received from the inside are not indigenous. For instance, patients so-called *garimpeiros*, working in clandestine gold mining, represent the majority of this group in our study. They are frequently dropped off by the dispensary with consciousness disorders. So, we cannot presume of the delay between the onset of the symptoms and the arrival to the health-care center. We can suspect not only an unidentified infectious trigger or even an emerging agent, but also toxic exposure or even toxics used in some traditional pharmacopeia. Over the “inside group,” the etiology was undetermined in half of the cases with a worst outcome. In addition, *Cryptococcus* was isolated in nine cases. Five of them were immunocompetent with no comorbidity. Dengue and Tonate viruses were isolated in one case each. Because of these differences, we think that the overview of the epidemiology of encephalitis in French Guiana needs to be split between those two areas of the study (inside and coastline).

The limits of our study are important. Our study was retrospective; so, we worked on data which were not originally collected for research. But, we recorded all the data through a centralized access from the hospital discharge database or from the patient’s files with a completeness rate of data collection at 96%. In addition, in a comparison of the outcomes of patients with encephalitis reported from prospective and retrospectives studies, Bernard et al.^39^ underline the few differences between the two processes of collecting data. Nonetheless, Granerod et al.^9,40^ pointed out the possible overestimation of encephalitis by clinical presentation mimicking encephalitis. In our study, after a first reading of the files and exclusion of cases without encephalitis, we corrected the risk of overestimation of the diagnosis by the reviewing of the patients’ files by two specialists to confirm the diagnosis and to assess the probability before the final inclusion. In our data collection, we did not put the stress on the etiological testing panel applied for the diagnosis. So, we cannot affirm that an exhaustive testing has been performed in cases with encephalitis with unknown cause or from autoimmune origin.

To the best of our knowledge, this is the first study from French Guiana and from Amazonian region reporting etiologic and prognosis factors in patients with encephalitis.

In addition, our hospital centralizes almost all serious patients of the department, notably by having the only ICU and the only infectious disease unit in French Guiana. For this reason, we think that the overview of the local situation is exhaustive because all patients with neurologic disorders or severe infectious conditions are transferred to our hospital. Furthermore, most of the specialists of the department are part of our hospital; so, almost all the follow-ups at 3 months were available in the database.

## CONCLUSION

Encephalitis in French Guiana is a life-threatening condition with a specific epidemiology. The most responsible infectious agent was *Cryptococcus* sp. in both immunocompetent and immunocompromised population. The myriad of etiologies found in our study reflects an already known epidemiology for some pathogens such as herpes simplex virus, varicella zoster virus, HIV, *S. pneumoniae*, or *Mycobacterium tuberculosis*. But the shape of the local epidemiology highlights the original infectious situation with pathogens such as *C. burnetii*¸ dengue virus, TONV, chikungunya, RABV, or *T. cruzi*. A focus should be placed on emerging triggers, especially in the population from the inside areas of the territory which has a significant poor outcome comparing with the population from the coastline. Predictive factors of poor outcome were coming from inside of the region, age older than 65 years, and need of mechanical ventilation. Further studies are needed to understand the specificities of encephalitis in the subgroups. Physicians should be aware from the specificities of encephalitis in the Amazonian region to prompt adequate screenings and antimicrobial treatments.
